# Impact of an outdoor loose parts intervention on Nova Scotia preschoolers' fundamental movement skills: a multi-methods randomized controlled trial

**DOI:** 10.3934/publichealth.2022015

**Published:** 2021-12-27

**Authors:** Karina Branje, Daniel Stevens, Heather Hobson, Sara Kirk, Michelle Stone

**Affiliations:** 1 School of Health and Human Performance, Dalhousie University, Halifax, Nova Scotia, Canada; 2 Department of Pediatrics, IWK Health Centre, Halifax, Nova Scotia, Canada; 3 Interdisciplinary Studies, Dalhousie University, Halifax, Nova Scotia, Canada; 4 Healthy Populations Institute, Dalhousie University, Halifax, Nova Scotia, Canada

**Keywords:** children, early childhood educators, health, outdoor play, physical literacy, fundamental movement skills

## Abstract

Development of fundamental movement skills in early childhood supports lifelong health. The potential for outdoor play with loose parts to enhance fundamental movement skills has not been investigated. A multi-methods randomized controlled design was used to determine the efficacy of integrating outdoor loose parts play into Nova Scotia childcare centers (19 sites: 11 interventions, 8 control). Movement skills (n = 209, age 3–5 years) were assessed over a 6-month period to investigate changes in fundamental movement skills over time and between groups. Qualitative data was also collected on the educators' perceptions of outdoor loose parts play. Quantitative data (fundamental movement skills) revealed a non-intervention effect, however, educators spoke of outdoor loose parts play providing opportunities to combine/ repeat movements and take risks; supporting physical, cognitive and socio-emotional (holistic) development; and increasing awareness of children's physical development and how to support it. Our findings demonstrate value in outdoor loose parts play for the development of fundamental movement skills in childcare settings.

## 1. Introduction

Play, particularly unstructured, self-directed, and/or free play, dominates early childhood, and is critical for physical, cognitive and socio-emotional development [1],[2]. Play is therefore critical for early learning and development that it is recognized in the United Nations Convention on the Rights of the Child [3]. Play fosters imperative skills such as problem solving, collaboration and creativity [4], offers opportunities for exploration and risk and contributes to self-awareness, confidence, competence, independence, perseverance, resilience, and positive mental health [5]. As children take risks in play, they master situations and challenges and develop perceptual motor skills and spatial-orientation abilities [6]. Outdoor play typically offers more affordances for diverse types of play, including risky play, and has unique contributions to children's overall growth and development [7].

Although the benefits of outdoor play are known, literature has noted marked historical declines in children's outdoor play in Canada and worldwide [8], leading Canada to promote strategies such as the Position Statement on Active Outdoor Play [9] and Outdoor Play Canada (https://www.outdoorplaycanada.ca/) that aim to increase children's active outdoor play. Consequently, there has been growing attention into how best to support children's active outdoor play, with its associated risks and benefits.

The childcare environment has a significant impact on the health and physical activity (PA) behaviors of young children [10]. A large number (~60%) of Canadian preschoolers attend licensed childcare centers [11] and spend a significant portion of their day there [12], providing an ideal setting for intervention. Systematic reviews have reported that intervening in childcare centers can have a positive impact on both children's PA [13] and their fundamental movement skills (FMS) [14]. FMS are the building blocks of movement and provide the basis for lifelong movement [15]. FMS include locomotor skills, object control skills, balance, agility, and fundamental sport skills [16],[17]. FMS are one component of physical literacy, defined as “the motivation, confidence, physical competence, knowledge and understanding to value and take responsibility for engagement in physical activities for life” [18]. Physical literacy is a concept that has been gaining increased attention internationally for its contribution to development, health and wellbeing in the early years and throughout the lifespan [19]. Children experience rapid brain growth and neuromuscular maturation in early life [20], making this period ideal for developing key locomotor, manipulative and balance skills, which form the basis for more complex movements and present an opportunity to engage in diverse PA. The early years represent a time where health behaviors are established, particularly PA, which tends to track throughout the lifespan [21]. If children do not become competent and confident movers in early life, they may start to disengage from PA in later years [22]. Developing FMS at a young age also has important health implications: it has been associated with a lower body weight [23],[24]; and, low FMS has been related to both low cardiorespiratory fitness and PA levels [25]. As such, the development of FMS in the early years can be seen as the gateway to lifelong PA participation and optimal health and wellbeing.

Several interventions have introduced structured, active play as a way to enhance young children's FMS [20],[26]–[28], some in childcare settings [20],[26], with several [26]–[28] showing significant improvements in children's FMS after exposure to active play. To date, no study has investigated the impact of unstructured, child-led outdoor play with loose parts on children's FMS.

Loose parts are open-ended play materials (manufactured or natural) that are moveable and non-dictated, allowing children to use them in creative ways [29]. Some examples include tires, tree stumps, wooden planks, rope, buckets and balls. Play with loose parts, in indoor and outdoor environments, has been gaining attention due to increased understanding of its potential to enhance multiple developmental domains in childhood [30]. Scholars are now advocating for children to be exposed to outdoor play environments that have a variety of loose parts available [31]. While there is some evidence on the value of outdoor loose parts play in enhancing aspects of children's health [30], most of the studies have been conducted with school-aged children and youth, and indicate an impact on children's socio-emotional [32] and cognitive [31] health, rather than their physical health, a limitation that has been noted in the literature [33]. Specifically, little evidence exists on the effectiveness of integrating loose parts into the outdoor environments of early years settings (e.g., home, childcare) as a means of improving PA and FMS [33].

Although previous childcare-based FMS interventions have been performed [34],[35], these have typically involved structured activity/play and have not included multi-methods (e.g., quantitative and qualitative data) in their designs. It has been noted that there is a need for additional high-quality outdoor play interventions in order to determine if it is an effective strategy for improving children's FMS [36]. To the best of our knowledge, there are no randomized controlled studies that have investigated the efficacy of outdoor loose parts play on the development of children's FMS. Furthermore, no study has explored the perspectives of early childhood educators in terms of the value of outdoor loose parts play for FMS development. Educators play a critical role in influencing the outdoor play environment, dictating how the space is allowed to be interacted with and shaping the way that children play [37]. As such, educators have a direct influence on children's opportunities for outdoor play, and also the quality of play experiences. The involvement of educators has also been identified as crucial to improving early childhood educational programmers [38].

Understanding educators' perspectives related to outdoor loose parts play, and the value this type of play may bring in terms of children's FMS development, could influence the adoption of outdoor loose parts play in other childcare settings as a mechanism for supporting key components of development, health and wellness in the early years. Therefore, the purpose of the present study was to explore the impact of a childcare-based outdoor loose parts play intervention on NS preschoolers' FMS using a multi-method, clustered, randomized control trial (RCT) design.

## 2. Materials and methods

### 2.1. Study design

This study is part of a larger cluster RCT, the Physical Literacy in the Early Years (PLEY) project [39]. The PLEY project focused on improving physical literacy, PA and active outdoor play in Nova Scotia preschoolers aged 3 to 5 years through the integration of loose parts into the outdoor environment of regulated childcare centers [39]. Additional objectives were to examine the impact of the project on improving educators' attitudes, beliefs, perceived competency and intentions toward incorporating outdoor loose parts play into practice; and the impact of the project on educators' and parents' understanding of play in child health and development. All Nova Scotia childcare centers serving children aged 3−5 years, with an enrolment greater than 20 children, were sent a general inquiry of interest. Centers that expressed interest were visited to meet with the director, further discuss the project, discuss and complete a survey, and view/photograph their designated outdoor play space and adjacent spaces if applicable. For further information on recruitment, see Houser and coworkers (2019) [39]. The intervention included 19 sites (intervention: n = 11; control: n = 8) and ran over a six- to eight-month period. The study took a staggered approach to the recruitment of childcare centers. Sixteen sites (8 intervention, 8 control) were recruited as part of the original cohort, and three additional intervention sites were added as a second cohort a year later to account for the drop out of one center and participant (child) withdrawal. Data collection took place from April 2016 to September 2018. The study was given full ethical approval from the Dalhousie University Research Ethics Board (REB 2016-3924) and registered as a trial with BioMed Central (ID# ISRCTN14058106); it also adhered to the CONSORT guidelines for randomized trials [39].

### 2.2. Intervention

After baseline data were collected, participating childcare centers were randomly assigned as an intervention or control site. Educators from intervention sites received training on: the importance of unstructured, child-directed active outdoor play for children's development, health and wellness, and the value of loose parts for supporting this type of play; FMS and the concept of physical literacy; and the value of risky, outdoor play for children's development, health and wellness. Educators had the chance to consider and describe photos of children engaging in outdoor loose parts play and to “play” with loose parts in an outdoor space. Finally, educators were given training on the importance of photographing children's outdoor loose parts play throughout the intervention; and on describing the play experiences, their contribution to children's FMS and physical literacy, their own role in supporting the play, and the level of risk associated with the play. The loose parts kits were then distributed to educators from intervention sites on completion of the training. The loose parts kit included buckets and lids, rope and a pully, tree cookies, milk crates, a package of hose tube, 20+ balls of a variety of sizes and weights, wood pieces, bread tray, large cardboard tubes, funnels of different sizes, a tarp, 5′ planks, 5′ PVC tubing (4′ and 2′ diameter), rocks, and tires [39]. The educators were asked to integrate the loose parts into childcare center outdoor spaces so they would always be available to the children. This ensured that children were exposed to the loose parts, and had the opportunity to play with them, when in the outdoor space. The control sites were asked to continue their planned outdoor play schedule and activities. All children between the ages of 3 to 5 years from participating centers were eligible to participate in the study. However, only children whose parents gave informed consent were formally assessed. Further details on recruitment and the intervention are provided elsewhere [39].

### 2.3. Data collection

#### 2.3.1. Children

Demographic information and anthropometric data were collected from participating children. Preschoolers (109 males, 88 females) had a mean age of 3.8 years at baseline (range = 3−5 years) and attended childcare centers that varied according to SES (see Table 1). According to the Canadian Society for Exercise Physiology (CSEP) protocols [40], height was measured in cm (to the nearest 0.1cm) using a portable stadiometer (SECA, Hamburg, Germany) and weight was measured in kg (to the nearest 0.1kg) using a digital scale (A&D Medical, Milpitas, CA, USA). Height and weight were used to calculate children's BMI (kg/m^2^; baseline: mean = 16.07, range = 12.73−20.88). The Test of Gross Motor Development-3 (TGMD-3) was used to evaluate children's FMS [37]. The TGMD-3 is a validated tool for assessing gross motor ability development in children aged 3 to 11 years [41]. A sum of all locomotor skills (run, hop, gallop, skip, horizontal jump and slide) and object control skills (one-hand strike, two-hand strike, dribble, catch, kick, underhand throw, overhand throw) was used to calculate a total gross motor score (total FMS score); the sum of all locomotor skills (total locomotor skills score) and object control skills (total object control skills score) were also derived (n = 178). As FMS are classified as locomotion, manipulation, and balance skills, it is a major limitation that the TGMD-3 only assesses locomotion and object manipulation [41]. Balance is a critical aspect of FMS, encouraging both fine and gross motor skills [42], and therefore a tool that assesses balance specifically would provide important information on this fundamental physical skill. For this reason, balance was additionally assessed using the Preschooler Gross Motor Quality Scale (PGMQ), a validated tool that includes four balance measurements (single leg standing, tandem standing, walking line forward, and walking line backward) [43]. A total balance score (sum of four balance measures) was calculated (n = 177). The researcher assessed one child at a time by first demonstrating how to correctly perform the skill and then asked the child to perform the skill. Each skill had various criteria (3−5 unique subscales) and was either scored a zero, representing a movement performed incorrectly, or one, representing a movement performed correctly. Each child was given one practice trial and two scored test trials for each skill. The two scored test trials were then added to get the total score for each skill. The possible scores vary between each dependent variable based on the number of possible points a child could score on each assessment. Possible locomotor scores range from 0−46, object control scores range from 0−54, total FMS scores range from 0−100, and balance scores range from 0−36. Each trained evaluator assessed one child at a time, scoring the multiple performance criteria for each skill, and providing a total score. The evaluator demonstrated how to correctly perform the skill to the child, and then asked the child to perform the skill. Children had one practice trial, followed by two scored trials. Study measures were collected at baseline (prior to intervention) and following the introduction of loose parts to intervention sites (3- and 6-month time points) in all sites (intervention and control). It was not possible to ensure data collectors were blinded to group assignment, as data were collected at the centers, where the loose parts were located.

#### 2.3.2. Educators

Educators (including several childcare directors and managers) participated in focus group sessions mid-way through the intervention (3 months; n = 24) and again at the end of the intervention (6 months; n = 27). For the purpose of this study, qualitative data were only collected from intervention sites. Participants were divided into small groups (3−4 per group) to ensure there would be opportunities for sharing between centers. The focus group question period lasted approximately 45−60 minutes, comprising mostly of in-depth conversations about the outdoor loose parts intervention. Educators provided photos of outdoor loose parts play and associated documentation to the research team ahead of focus group sessions, and shared these as a way of prompting discussion on the contribution of outdoor loose parts play to children's FMS and physical literacy, the level of risk associated with this type of play, and their own role in supporting the play. At each focus group table, facilitators described the purpose of the focus groups, provided guidelines ensuring privacy and confidentiality, and received verbal consent from each educator to audio-record the conversations and to use anything educators said as anonymous quotes. Focus group questions were divided into the following categories: active outdoor play, loose parts, risk-taking, policies, and challenges/benefits of the intervention.

### 2.4. Data analysis

#### 2.4.1. Quantitative

All quantitative analyses were conducted using Statistical Analysis Software (SAS) (Version 9.4). A priori sample size calculations for the four dependent variables (total FMS score, total locomotor skills score, total object control skills score and total balance score) with an observed effect size of Cohen's d = 0.8 and probability level of 0.05, indicates that sample sizes ranging from 106 to 144 total participants would be required for sufficient statistical power to prevent the probability of a type-II error. Prior to statistical analysis the Shapiro-Wilk test was used to determine whether data were normally distributed. All data were normally distributed prior to analysis. Descriptive statistics of children's demographics, BMI, and the dependent variables for both control and intervention groups were used to describe participants. Chi-square analyses were used to explore any differences in distribution of independent variable characteristics between groups. Similar to Adamo and colleagues (2014), [26] in order to include all TGMD-3 [41] and PGMQ [43] assessments, an intention-to-treat basis was used. Linear multilevel modelling for repeated measures was used to determine if children at intervention sites had greater increases in TGMD-3 [41] measured total FMS scores, and subscales of the total score (total object control skills score, and total locomotor skills score), and PGMQ-measured total balance scores [43] over time compared to children at control sites. The data set was hierarchical, with time being the first level variable, the children being the second level variable, and the center being the third level variable. Multilevel modelling accounted for possible clustering of childcare center, as children who belong to the same center may have been more similar. Possible confounding variables, such as age, sex, BMI, environment and SES were also included in the models, as previous literature has shown these variables influence children's FMS [44]. 95% confidence intervals (95% CI) and p-values were calculated and statistical significance was defined as an alpha less than 0.05.

#### 2.4.2. Qualitative

Focus group data were transcribed verbatim and organized using Microsoft word (version 16.16.3). Qualitative data were analyzed using thematic analysis on Microsoft Word (version 16.16.3), allowing the researcher to describe the data without over-interpretation, and instead use the words of the participants [45]. An inductive coding process was used, which is a common form of analysis for understudied topics [45]. The researchers chose themes that both occurred frequently and answered the research objectives [45]. Qualitative description was used to summarize the information from the data and present it in a clear way [46], allowing the educators' perceptions of the outdoor loose parts play intervention on children's FMS to be determined.

## 3. Results

### 3.1. Quantitative

Table 1 presents the descriptive statistics (number of observations (N) and the percent of number of observations (%)) for the categorical independent variables at baseline. Group, sex, and environment were well balanced; however, age was unbalanced, with the 4-year-olds comprising 50% of the sample. SES was originally collected in three categories. However, the low SES category comprised 45% of the sample, and therefore the medium and high categories were collapsed to make it more balanced. Seven unrealistic low or high BMI values were removed from the dataset. Chi-square analysis revealed there were no significant differences in the distribution of baseline characteristics between control and intervention groups.

**Table 1.** Descriptive statistics for independent variables by group.

Independent variable	All sites = 19	Intervention (sites = 11)	Control (sites = 8)
Environment (n = 209)			
Urban (n, % of all sites)	76, 36.36	39, 51.31	37, 48.69
Suburban (n, % of all sites)	62, 29.67	39, 62.90	23, 37.10
Rural (n, % of all sites)	71, 33.94	37, 52.11	34, 47.89
Socio-economic status (n = 209)*			
Low (n, % of all sites)	94, 45.00	51, 54.25	43, 45.75
Moderate & High (n, % of all sites)	115, 55.00	64, 55.65	51, 44.35
Age (n = 195)			
3 years (n, % of all sites)	66, 33.85	39, 59.09	27, 40.91
4 years (n, % of all sites)	96, 49.23	52, 54.16	44, 45.84
5 years (n, % of all sites)	33, 19.92	19, 57.57	14, 42.43
Sex (n = 197)			
Male (n, % of all sites)	109, 55.33	63, 57.79	46, 42.21
Female (n, % of all sites)	88, 44.67	41, 46.59	47, 53.41

*Note: No significant differences (p > 0.05) in distribution (Chi-square) of independent variables between intervention and control sites. To determine low and moderate-high SES, childcare centers were first geocoded, according to their civic address, to provide a latitude and longitude, which was used to determine the dissemination area for that location. Median household income was assigned to each center, using Census data. Sites were grouped according to tertiles initially, based on median household income, and collapsed into two categories (low and moderate-high SES) to provide a more even distribution of the data.

Table 2 presents the mean, standard deviation and range of the four FMS variables (total FMS score, total locomotor skills score, total object control skills score, total balance score) across the three time points (baseline, 3- and 6-months) in both the intervention and control sites. All of the FMS variables have mean values that increased over the three time points (1 = baseline, 2 = 3-months, 3 = 6-months), regardless if the site was intervention or control.

While all four dependent variables changed significantly over the three time points (baseline, 3- and 6-months); multilevel modelling showed that adding group (intervention and control) did not decrease the -2LL by ≥ 3.84 (based on the chi-squared distribution) and, therefore, it did not make any of the models better at predicting the dependent variable. Furthermore, multilevel modelling showed group to be non-significant in each model (total locomotor skills score, p = 0.99; total object control skills score, p = 0.18; total FMS score, p = 0.51; and total balance score, p = 0.08). The outdoor loose parts play intervention, therefore, did not predict any of the four dependent variables.

**Table 2.** Descriptive statistics (N, Mean, Std., Range) of the dependent FMS variables by time.

	Baseline			3-Month			6-Month		
	N	Mean (std)	Range	N	Mean (std)	Range	N	Mean (std)	Range
Locomotor
Control^b,c^	75	25.17 (8.18)	6.00−43.00	44	26.16 (7.04)	15.00−44.00	36	29.33 (8.99)	9.00−42.00
Intervention^a,b,c^	103	24.60 (9.24)	5.00−46.00	69	27.74 (9.62)	5.00−46.00	63	30.89 (7.08)	16.00−46.00
Object Control
Control^a,c^	75	22.33 (6.92)	6.00−39.00	44	27.78 (6.40)	12.00−42.00	36	29.06 (7.10)	14.00−47.00
Intervention^a,c^	103	24.66 (8.20)	8.00−44.00	69	28.43 (8.43)	13.00−50.00	63	30.22 (7.86)	14.00−53.00
Total FMS
Control^a,b,c^	75	47.51 (12.91)	14.00−77.00	44	53.96 (11.81)	27.00−78.00	36	58.64 (14.46)	31.00−85.00
Intervention^a,c^	103	49.26 (15.48)	17.00−81.00	69	56.32 (8.43)	21.00−96.00	63	60.32 (12.48)	34.00−63.00
Balance
Control^b,c^	74	15.28 (7.05)	0.00−30.00	44	17.78 (6.98)	5.00−34.00	36	21.25 (8.11)	2.00−35.00
Intervention^a,b,c^	103	15.90 (7.67)	0.00−33.00	69	19.39 (7.13)	5.00−35.00	63	21.05 (6.26)	2.00−34.00

*Note: locomotor = total locomotor skills score; object control = total object control skills score; total FMS = total FMS score; balance = total balance score.

a = significant change between baseline and 3 months in groups (p ≤ 0.05)

b = significant change between 3 months and 6 months in groups (p ≤ 0.05)

c = significant change between baseline and 6 months in groups (p ≤ 0.05)

### 3.2. Qualitative

By exploring the focus group discussions at 3- and 6-months, three themes, and several sub-themes, were developed in relation to educators' perceptions of the intervention on preschoolers' FMS. These themes were: movement skill development; holistic development; and educator development. Each theme is supported by quotations from focus group participants, identified using the 3-month (3M) or 6-month (6M) time point and identified by either original cohort (OC) or new cohort (NC). Several steps were taken to ensure trustworthiness and rigor in the study's qualitative analysis. Indeed, focus groups were facilitated to allow participants to speak freely, ensure their voices were heard, and accurately capture the participants' perceptions of the outdoor loose parts play intervention; this was achieved by encouraging all participants to speak when they felt comfortable, by asking all participants to respect each other's opinions, and audio-recording and transcribing the focus groups verbatim. Finally, the participants' direct quotes were reported to ensure no misinterpretation [47].

*Theme 1: Movement skill development*. Through focus group discussions, it became evident that educators felt the children were developing FMS through outdoor loose parts play. Educators spoke of various factors that influenced FMS development: the combination of movements; repetition; and risk taking.

*1.1. Combination of movements*. Educators explained that when children were playing with the loose parts, they were moving their bodies in a variety of ways. These descriptions of children's outdoor loose parts play highlighted multiple FMS (e.g., balancing, pulling, carrying), rather than just one FMS in isolation. Children needed to execute a series of FMS in order to move and manipulate the loose parts, “lifting, balancing, pulling and carrying, some bending, some stretching going down to get the balls and then crawling over to get them.” (6M-OC)

They would have to, you know, crawl along, on their hands and knees and then shimmying underneath and climbing through, like this, on their stomach, and um, lifting, dragging, rolling, they were doing everything to try and manoeuvre these [loose parts]. (3M-NC)

One educator spoke about observing a change in how the children were moving their bodies. They expressed that, prior to the introduction of loose parts into the outdoor play environment, children would be only running, or only jumping, executing just one FMS in isolation. Once loose parts were introduced, the children's play was expanded and their movement patterns became more complex and exploratory, with a variety of FMS emerging within one activity.

*1.2. Repetition*. Educators often spoke about children using the same loose parts to build similar projects over and over. This repeated use of the same loose parts allowed children to unintentionally practice the FMS associated with each project. Educators recalled the children learning from their mistakes (e.g., falling off a balanced wooden plank), and then recreating and extending their play with the loose parts. By repeating the movements associated with each task, the children were provided with the opportunity to learn from their mistakes and develop their FMS. Educators recalled the children developing confidence after completing the FMS a number of times, eventually leading to the children progressing in these skills.

Giving them the opportunities to learn from their mistakes cause they are going to make mistakes and chances are they are going to fall, and chances are they might hurt themselves but they're also going to learn the next time I'm not going to do that so how can I make it better so that doesn't happen again. (6M-OC)

Educators also spoke about how children's progression of FMS coincided with increased confidence. As children repeated their movements, educators felt they became more confident that they could complete the movements, helping to improve their associated FMS.

Some of them don't have the balance or coordination that some of the older ones do, so they do start up a little more slowly and tentative then – but then when they go through these movements – you can really see that their coordination is improving, their balance is improving, and they – their fear is gone now. (3M-NC)

An educator from one site relayed a story about a child initially being cautious to walk across a wooden plank a few feet off of the ground; after attempting to traverse it, and being successful, the child's confidence rose, as did their motivation to move across it and this little girl is cautious so when the children at first built this little ramp, probably two and a half feet high off the ground. She was very cautious and the little boy that first built it he was encouraging her, you can do it, you can do it, and he actually would walk along holding her hand until she became comfortable. And then it's hard to tell but she's running across it, she moved on from nervous to not cautious at all. (6M-OC)

Educators also spoke about how the children would often reproduce what they had previously made on a different day or in a different area of the playground. By reproducing what they previously made with the loose parts, the children were provided with the opportunity to build upon the same FMS they used before. Several educators mentioned that when the children were repeating their play experiences with the loose parts, they often extended it by adding additional loose parts, having more children join the play, or by moving their bodies differently. This was perceived to allow the children to continue their play, and gave them the opportunity to try new movements and build upon previous FMS. Educators indicated that by extending their play, the children were able to challenge themselves, which maintained their interest and kept them playing with the loose parts.

Sometimes they need the challenge. Like if they've climbed or ran or balanced on this thing like 90 times a day, they need a new way. They still want to do a bit of exploring so it's like I'm going to walk around the perimeter of our play area backwards today, it's still balancing but now I'm doing it backwards cause that's new, and I've tried that, can I do that? I don't know. So, it is like the sense of accomplishment if they can do it. (3M-OC)

*1.3. Risk taking*. As the educators discussed the children engaging in risky play with loose parts, it was evident to the educators that the children were also developing their FMS. Playing with loose parts in their outdoor environment seemed to allow children to test the limits of what their bodies could do; it also presented an opportunity for the children to develop risk-assessment skills. The educators recalled how children's movement and manipulation of loose parts sometimes created an element of risk (e.g., carrying heavy loose parts, climbing and balancing at great height). In order to navigate these risks, children needed to move their bodies in different ways, which demanded various FMS.

This boy carrying three crates is definitely risky because like you just said, it could drop, he's holding on to the bottom crate and the other two are just there, so he is balancing and he can't really see, he can see a little bit cause there's holes but it's pretty risky, and he's walking on uneven ground which is [inherently] risky cause he could trip, he could drop the crates. And then the one with the slide and the rope that's risky cause they could get a burn on the hands from the rope, they could slip when climbing up. (3M-OC)

One educator spoke about how children's engagement with loose parts offered challenges (and inherent risks within these challenges) that children were determined to conquer, which demanded diverse movements. In this instance, the educator recalled how amazed they were of the child for being able to complete the task, noting how risky and difficult the task was. By allowing the children the opportunity to engage in risky play, the educators also afforded the children the opportunity to develop their FMS.

They climbed up on with the rope and got on to the tree but the risk was kind of medium so I stood behind them because if they fell it was you know quite a distance but the determination to get their foot over the tire, to hold themselves up with the rope, to touch the tree and hold the tree was amazing. (3M-NC)

*Theme 2: Holistic development*. The educators often spoke about the children using cognitive and socio-emotional skills while moving their bodies to engage with the loose parts. Not only did educators perceive that children were developing various FMS through outdoor loose parts play, but as that was happening, they shared examples of how children were developing cognitive and socio-emotional skills such as problem solving, mentoring and teamwork, imagination, communication and enjoyment.

*2.1. Problem solving*. Educators described how children often had to problem solve while playing with the loose parts. Several educators spoke about children needing to figure out how to move their bodies differently with each loose part, as well as with one another. Examples educators used of the children problem solving were the children figuring out how to fit a pipe in a hole, and problem solving how to throw a plastic ball differently due to the weather conditions.

Because they were harder to throw because they were so light and the wind would kind of, so they had to sort of adjust the course of where they were going to throw it if it was a windy day. So, yah that kind of took a different skill, different maneuvering. (3M-OC)

One educator described a specific photograph where several children were using the loose parts as an obstacle course. One child, however, wanted to go in the opposite direction of the other children. The educator used the photograph to describe how the child had to problem solve in order to get around the other children without falling or disrupting the other children's play. The child had to figure out how to move their body in order to accomplish their goal of going through the obstacle course backwards.

They were trying to figure out how to get around each other because they were like the rest of them were kind of heading this way and he was trying to go the opposite way as everybody else, so he was trying to figure out how to get around here without falling off in the end he ended up getting, he got down into the sandbox and then climbed back up on the other side of her and kept going, and just kept walking around children every time he got to them, he would get down and then back up. He was determined to go backwards. (6M-NC)

*2.2. Mentoring and teamwork*. Several educators recalled that the children often made creations that involved an element of risk, such as bridges and teeter totters. Educators indicated that these projects were frightening to many of the younger children who might not have experienced them before. The older children recognized the younger children's fear, and offered support and guidance to them, which helped to both ease the younger children's anxiety and build their confidence. Educators spoke about how the older children taught the younger children the necessary movements for the specific task and/or helped them along the way by guiding them or holding their hands. By showing the younger children how to complete the movements, they were helping to develop these children's FMS, while also building upon their own FMS.

So when they built this and the other younger ones were standing back watching them, you could just kind of see fear in their eyes and so they thought o.k. well I'll give it a try or whatever and some of them were really wobbly, they didn't have the coordination that these guys had at all and in fact some of the other children would come over and take their hand and show them around and then you could see the confidence building each time they went till eventually they could do it themselves. So, it was really nice to see how they worked together and were mentoring each other and cooperating and helping the younger ones, so that they could grow their gross motor skills too so I think that was one big thing, advantage of the whole program right because they, the younger ones learned from the older ones yah. (6M-NC)

As many of the loose parts are larger than the children in this study (e.g., wooden planks, PVC pipes, tires), educators spoke about the children having to work together in order to move and manipulate the objects. By working in teams, children were able to communicate how they were going to use these objects. Working in teams also demanded that children coordinate their own bodies with one another.

When they were building it, some of the children were just like let's make it bigger, and if one child would be carrying one of the big planks, they would kind of yell over like somebody come help cause it's too big, it's too heavy, and they would kind of partner up and work together. (6M-NC)

*2.3. Imagination and creativity*. Educators spoke about children using their imagination to play with the loose parts in different ways, which meant that they had to move their bodies in a variety of different ways. Whether the children were building a rocket ship, a campfire, or an obstacle course, they had to more their bodies differently in order to build each one.

At [learning center] I noticed there was, it definitely increased the creativity in their play it seemed to increase their movement because all of a sudden they weren't just o.k. I'm going to play here at the slide the whole time we're outside, it was you know they were creating all these different games and imaginary play and that was getting them to use a lot more of our playground space like o.k. we have to go over here and we have to get this and then we need to carry all of that over here because this is where our super hero base is and then we have to go over here and save this person. So, they were incorporating it into imaginative play a lot and then I felt that encouraged them to be moving around more. (3M-OC)

Educators also spoke about how children would find new ways to move with the loose parts, which ended up enhancing their balance and coordination; and also increased the level of risk associated with the play. “With the loose parts, it encourages creativity so there's so much they can do with them. the riskiness could increase as they become more creative and they think of different ways to play with the loose parts too.” (3M-NC)

*2.4. Communication*. The educators recalled how the children started to communicate with one another much more when they were playing with the loose parts. By using more words, the children were able to share their ideas and plan what they wanted to do with the loose parts, which affected how they moved the loose parts and their own bodies.

So, they decided that they wanted to make a bridge, so they put the ramps on here and so the fascinating thing about it is that they were moving this board because they, in talking to each other and experimenting with movement, they began to realize that if it was inclined down that the truck would go down, and if they put it level the truck would go straight and if they lifted it up, the truck would go backwards. And so, they were discussing this and all the different ways that they were controlling the movement of the truck. (6M-NC)

One educator also spoke about how the opportunity to play with and manipulate loose parts outdoors strengthened children's communication skills, particularly those whose first language was not English. The children had to communicate in English in order to participate in the play, moving the loose parts and their own bodies.

We have a lot of children at this site that are learning English it's been remarkable like how fast these children are learning English because they're trying to help with building these things and so they're learning the words for it and they'll say can I help and then listening to all the cues that they're giving each other and catching on to them until they're using them. (6M-NC)

*2.5. Enjoyment*. While describing their experiences during the intervention, the educators often mentioned how the children were laughing and smiling while physically playing with the loose parts. The children were happy that they had a larger variety of materials to play with, and the additional freedom to explore their outdoor environment. Educators spoke about how children seemed to get joy and pleasure out of physically manipulating the loose parts and tended to laugh and smile a lot while imitating each other's movements.

I think they were a lot happier, I mean seeing all the same stuff on the playground every day and then getting all of this stuff that they could just put together themselves. I find them busier and I find them much happier than just having the climber and the soccer net or whatever. (3M-NC)

*Theme 3: Educator development*. When educators were asked about their own experiences with the loose parts, many revealed the outdoor loose parts intervention made them more aware of children's physical development and their physical skill sets (i.e., FMS); as well as how to support children's physical development.

*3.1. Awareness*. Educators recognized that they were much more knowledgeable about, and comfortable discussing, children's cognitive and socio-emotional development, and how to support it, rather than their physical development. Multiple educators spoke about how they saw the children moving in different ways when they played with the loose parts, and how perhaps they would not have been so aware of the children's movements had the intervention not made them focus on the children's physical development. However, these results were obtained from the educators' own perspectives and, therefore, one needs to be careful not to generalize to the overall population.

I think it helped me focus more on the physical and the gross motor skills where it is so easy when you see children engaged with loose parts to see like the cognitive benefits and the social-emotional, and those come to mind so easily and we get so excited about them but it's really helped me focus more on what the physical skills are and what skills they need to work on, what they're already really good at so I feel like I'm better at identifying that now. (6M-OC)

One educator recognized that outdoor loose parts play was benefitting the children by increasing their physical exertion. When the children were using more energy to move and manipulate the loose parts, they were able to calm down inside. Educators noted that prior to the introduction of loose parts, the children had not burned off enough energy outside to calm down inside, making it difficult for them to engage in quiet time.

*3.2. Support*. Many educators spoke about how they have changed the way in which they support children's FMS development. Several educators mentioned that once the outdoor loose parts play intervention began, they became more cognisant of the language they were using around the children. This change in attitude allowed educators to step back and allow the children to evaluate the situation themselves, instead of stopping them when there might have been an element of risk and I found I've been trying to steer away from be careful and kind of phrase like more open-ended questions, like what would happen if you step your foot there. So, I definitely try and encourage the children to test their own limits of self-risk and their comfortableness in their risky play. (6M-OC)

Educators spoke about how their involvement in the project had increased their understanding of how to support children's physical development through outdoor loose parts play, “supervise, and let them play and experiment with their gross motor skills, and take risks, if they want to, and jump from wherever they want and balance where they want.” (3M-NC)

Finally, one educator mentioned that when educators (in general) recognize that being outside is beneficial to children's physical development, they will continue to be aware of the physical domain and will become more comfortable supporting it. They remarked that when educators “realize that we truly can help the children develop certain skills that have to do with their bodies and how to use them, then we won't necessarily overlook that, which I think is super important.” (3M-NC)

## 4. Discussion

Despite quantitative data showing a non-intervention effect (e.g., no statistically significant difference in FMS between the intervention and control group), qualitative data presented positive observations by educators on the value of outdoor loose parts play. These included providing opportunities for children to combine and repeat movements and take risks and, therefore, supporting physical, cognitive and socio-emotional development. If outdoor loose parts play can have the same effect as a structured play program on children's FMS, and also elicit a sense of joy, it can provide a means for encouraging children to engage in PA and sustain PA behavior.

Quantitative analyses revealed that children's grouping (intervention or control) did not have a significant effect on any the four FMS variables. In other words, children exposed to the outdoor loose parts play intervention had similar FMS to children in the control group across the study time points. Analyses revealed that time had a significant effect on all four FMS variables (total FMS score, total locomotor skills score, total object control skills score, and total balance score), all of which were significantly higher at the 6-month time point in comparison to baseline, as might be expected given development. Quantitative analysis also revealed that age had a significant effect on FMS scores, with scores increasing alongside of age. These results are in line with previous literature stating that as age increases, children's FMS also increase [14],[48]. These findings are not surprising, as children are provided with opportunities to build upon their FMS over time [14].

Our observations are not altogether surprising, given that the few childcare-based outdoor play interventions that focused on improving children's FMS have seen varied results, with some showing significant improvements in children's FMS after exposure to active play [27],[28], and others demonstrating a non-significant effect [20]. The varying results may be explained by differences between the active play session interventions. Johnstone and colleagues (2019) [28] explored an intervention involving 30 minutes of games and 30 minutes of free play, while an intervention by Foulkes et al. (2017) [20] involved 1 hour of warming up, dancing, jumping, accuracy games, and a cool down. Lastly, Tortella and colleagues (2016) [27] examined 30-minutes of unstructured play and 30-minutes of structured play on a gross-motor specific playground. A recent review notes there is large variability in the play opportunities provided to children, within these unstructured play interventions, as the methods themselves are quite different [2]. The differences in interventions and the varied results together highlight the ongoing need to explore the relationship between outdoor play (particularly unstructured outdoor play) and young children's FMS. The current study differs from the above-mentioned FMS interventions, as it involved unstructured, child-led, outdoor loose parts play each time the children were in their outdoor play environment, as opposed to a 1-hour intervention that included both structured and unstructured outdoor play.

Additionally, previous research assessing children's FMS have not used one consistent FMS assessment tool, and the interventions have differed in duration, making it difficult to compare results. Exploring other FMS assessment tools may help to provide a potential reason as to why there was no change in quantitative assessments of the children's FMS. The TGMD-3 and PGMQ assessment tools assess whether a child can perform the skill correctly, using a 0 or 1 classification [41],[43]. There are currently other validated and reliable tools, such as the PLAY Tools, which score a child using a range of 1−100 [49]. The TGMD-3 and PGMQ assessments are far less sensitive to change in children's FMS when comparing them to the PLAY Tools. For example, the TGMD-3 and PGMQ will only show a positive change if the child could not do the skill at baseline, and then improved to a point where they could do the skill. To date, children's FMS are typically assessed quantitatively. Without qualitative data, researchers are missing context and depth on how children are playing, and the benefits it has to their FMS and other aspects of development.

Through focus group discussions, it became evident that educators felt that outdoor loose parts play was benefiting children's FMS development, along with other aspects of their health and development. When educators were describing the children moving, they frequently spoke of the children using a combination of FMS. Flannigan and Dietze (2018) [31] similarly found that children would move their bodies in a variety of different ways when manipulating the loose parts, stating how “children would jump and walk and squat over a pile of logs” (Flannigan & Dietze, 2017, p4) [31]. This is an important finding in relation to the quantitative results. Educator focus group data suggest that children were not engaging in many of the movements that the TGMD-3 [41] and PGMG [43] assessed. This finding may explain why children exposed to outdoor loose parts play had similar FMS scores to children at control sites. Children may have not improved at individual skills such as running, hopping, and throwing independently, as they were not doing these individual skills while playing with the loose parts; they were instead doing skills such as lifting, pulling, carrying, bending, and stretching. Educators also described how children were using various combinations of FMS; neither the TGMD-3 [41] nor the PGMQ [43] are designed to assess how FMS are used in combination with one another. Additionally, educators did not describe children running with loose parts, throwing balls, or kicking balls, all of which were skills assessed with the TGMD-3 [41]. This may mean that the open-ended nature of outdoor loose parts play cannot be easily or reliably assessed using a structured FMS tool, but rather requires new ways to think about evaluating the development of FMS. Currently, we are not aware of a validated tool that can assess the movements educators witnessed while the children were playing with the loose parts, such as dragging, lifting, and pushing, all of which are important FMS.

Educators also highlighted how outdoor loose parts play provided an opportunity for children to repeat movements and consequently develop associated FMS. As children repeat the same skills over and over, they learn from their mistakes, become more confident in themselves, and learn to master skills. Children independently decided to repeat the movements they were using during their loose parts play, possibly mimicking the effect of structured play interventions that have been found to improve children's FMS [50]. Literature has shown that children acquire FMS when given the opportunity to practice and build upon their skills [25]. By providing children with ample time to play with loose parts outdoors, children had ample opportunity to move and manipulate these objects and thus develop their own FMS.

Educators also indicated that children became more confident in themselves after attempting a FMS a number of times, which is consistent with previous literature [38]. Gehris and colleagues (2014) [38] found that children were helping other children develop their movement skills and confidence by demonstrating the skills and then allowing the child to attempt them on their own, which is consistent with the mentoring and teamwork that emerged in the current study. These findings show the strong connection between educators' perceptions of children's confidence and FMS, and also educators' perceptions of children's motivation to continue to explore movements, all of which are components of physical literacy. This study additionally found that outdoor loose parts play allowed children to take risks, such as balancing, climbing at heights and carrying heavy materials; in doing so, children developed both risk-assessment skills and FMS. The value of risk taking to children's health and development is apparent [9],[51]. This study adds to the literature by showing that not only does outdoor loose parts play provide an opportunity for children to take risks and develop risk-assessment skills — it also supports their FMS development. This is an important and novel finding that adds to the growing body of literature on the value of unstructured, child-directed outdoor play for children's health and development.

Educators also revealed that outdoor loose parts play provided a means for holistic development, with children developing physically, as well as cognitively and socio-emotionally, as they moved and manipulated the loose parts outdoors and engaged with others. The physical benefits of loose parts play are largely understudied, with PA being the only variable investigated [32]. To date, the majority of literature has focused on children's cognitive [52] and socio-emotional [2],[31] development; and no study has revealed the value of outdoor loose parts play for supporting FMS and other aspects of development concurrently. Through outdoor loose parts play, educators observed that the children developed physical skills (FMS) while also developing their problem-solving skills and their mentoring and teamwork skills. In addition, educators reported that the children's imagination flourished, communication skills developed, and they enjoyed themselves. These findings demonstrate the real value of outdoor loose parts play for holistic development. The Nova Scotia Early Learning Curriculum Framework believes that children's learning is holistic in nature and encourages their educators to take a holistic approach to teaching [53]; findings from our study demonstrate the alignment of outdoor loose parts play with this approach. A notable finding from this study is the influence that outdoor loose parts play had on children's English communication skills for children whose first language was not English. The inclusive nature of loose parts may aid children who are struggling to learn a language as they can still participate in the play without being fluent, and also develop their communication skills while doing so. Similar to our study, Flannigan and Dietze (2018) [31] found an improvement in children's communication skills after being exposed to outdoor loose parts play, and an increase in pro-social behaviors such as leadership and cooperation while engaging with other children. Educators in the current study also recalled how often children were laughing while they were physically manipulating, and building with, loose parts. Keeping the focus on fun is important, as children are more likely to participate in an activity they enjoy. This finding provides rationale for the inclusion of outdoor loose parts play in future interventions, as children enjoy playing with them, and they elicit diverse developmental benefits.

Educators also spoke about how being part of the PLEY project increased their awareness of the importance of the children's physical development and how to support it. Educators indicated they learned how to focus on the physical skills children were developing while playing with loose parts outdoors. A recent study introducing loose parts play pop-up playgrounds into early childhood educator curriculum similarly found that when educators were exposed to children playing with loose part they were able to learn more about play and its importance in childhood development. Participating in loose parts play with the children enabled theses educators to observe and reflect on the children's play. The educators within the present study also stated that the PLEY project allowed them to become more confident in supporting the children's development of their FMS [54]. This is an important finding, as previous literature notes the critical role that educators have in promoting play during care hours, ultimately shaping the way children play [37]. As educators have such a large influence on children's play, it is critical they understand the importance of children's physical development and FMS, and the impact outdoor loose parts play can have.

### 4.1. Strengths

This study is novel in that it is the first to explore the impact of outdoor loose parts play on children's FMS, and is strengthened by the use of a clustered, RCT design and multiple data sources (quantitative and qualitative data), which allowed a deeper understanding of the research question. The study design and the prospective nature of the investigation helped minimize selection bias and afforded greater control during data collection at the daycare centers and focus group meetings. The design of the study also allowed causal inferences with the quantitative data (FMS) to be made providing the strongest empirical evidence of the intervention's potential efficacy. The use of a multi-methods, clustered, RCT design highlighted the discrepancies between quantitative and qualitative results, with quantitative analyses revealing that outdoor loose parts play did not significantly improve children's FMS beyond the control condition, and qualitative analyses revealing its value in children's FMS development. This is a notable finding and highlights the merit in collecting both quantitative and qualitative data to get a deeper understanding of the contribution of outdoor play to children's health and development. Few outdoor play interventions have included multi-methods approaches; the findings from this study demonstrate the worth of considering this approach in future work. Childcare centers from across Nova Scotia with varying SES and environments were included in this study, which enhances the diversity of the research and ensures the results are more generalizable. The inclusion of educators' perceptions and experiences of outdoor loose parts play and their relationship with children's FMS development is a novel contribution to the literature, and important given the profound influence of educators on children's development, health and wellness. Findings can be shared with other early childhood educators as a way of supporting outdoor loose parts play in other early years settings. However, these were the perceptions and experiences of the educators, and caution is needed when extrapolating these findings to the overall population.

### 4.2. Limitations

There are several limitations that may have impacted the findings from this research. The large dropout rate (44%) was one limitation. These sample size thresholds were achieved at the start of the study but decreased over the course of the investigation. Consequently, this may have affected the statistical power to detect group differences. Therefore, we recommend to those who intend to replicate our study, that they anticipate small effects and build an adequate sample size into their design to accommodate attrition. This study would have benefitted from starting data collection more than six months prior to the beginning of school (September) in order to minimize the number of preschoolers who withdrew from the study as a result of attending school in the fall. Another limitation of this study was the effect of season. The original cohort (n = 16 childcare centers) and new cohort (n = 3 childcare centers) were on different data collection timelines. Due to the seasonal differences, children may have had different opportunities to engage with the loose parts outdoors. Educators indicated that one of the major challenges of the study was the loose parts getting buried under snow in winter, limiting which materials the children could play with [55]. This may mean that children in the original cohort—the majority of the sample—were able to play with the loose parts more during the beginning of the intervention (spring) compared to the end (winter), possibly limiting their opportunities to develop FMS throughout the entire study. It is also important to note that researchers did not perform a manipulation check. Although experiences from the educators informed researchers that children were playing with the loose parts, there was no way to ensure all children participating in the study played with the loose parts. Another limitation was the inability of the TGMD-3 [41] and PGMQ [43] to adequately measure the various FMS children were developing through outdoor loose parts play (e.g., pulling, pushing, carrying, bending, stretching, lifting). Although another assessment tool may have been more appropriate within this study, determining that the TGMD-3 and PGMQ were not assessing the FMS children were using throughout this intervention is important information. This finding may influence the way researchers assess children's FMS in future interventions. Previous research has used video recording to analyze children's play behaviors [56]. By video recording the children, analyses can be more precise, as movements can be re-played, slowed down or sped up [57]. This method would have allowed researchers in the present study to assess how the children were playing with loose parts throughout an entire outdoor play session and what FMS were emerging through the play. An additional limitation is that total FMS scores were used as dependent variables in this study (total locomotor skills score, total object control skills score, total FMS score, and total balance score); and therefore the impact of outdoor loose parts play on specific FMS (e.g., running, hopping, galloping, catching, kicking) was not explored. The majority of previous literature has explored children's FMS in a similar way, using total FMS scores [20],[28],[58]–[60]. However, some studies have assessed the impact of interventions on specific FMS [27]. Examining the impact of outdoor loose parts play on specific FMS may help to determine the FMS that are most/ least impacted by outdoor loose parts play. While this was not the objective of the current study, this is something that could be explored in future.

## 5. Conclusions

The early years are a critical time for development, and play is fundamental to children's physical, cognitive, and socio-emotional development [1]. Determining the types of play that allow children to not only develop within one domain, but rather holistically in all domains, is critical for the creation of future initiatives aimed at improving children's development and overall health and wellness. Literature shows how opportunities for children to play outdoors have declined historically [8]; consequently, there is great interest in determining how to support children's outdoor play experiences and also enrich the quality of outdoor play. Although quantitative data did not show a significant effect of loose parts play on children's FMS development, qualitative data indicates that when children play outdoors with loose parts, it enables them to combine and repeat movements, and take risks, which supports overall FMS development. These inconsistent findings highlight the need for a validated tool that captures skills such as pulling, pushing, and lifting, all of which are FMS educators saw children perform while playing with loose parts. Outdoor loose parts play also allows children to develop holistically, strengthening their physical, cognitive and socio-emotional wellbeing. Importantly, our study revealed that educators are more comfortable in recognizing and supporting children's cognitive and socio-emotional development than their physical development. However, by participating in this intervention, educators became more knowledgeable about children's physical development and more confident in how to support it through outdoor loose parts play. These findings underscore the need for early childhood education curriculum and training to focus on developing educators' knowledge about physical development in early childhood and the value of outdoor loose parts play for supporting it. Finally, this study highlighted the impact outdoor loose parts play had on children's physical literacy as a whole (children's FMS, and also educators' perceptions of children's confidence and motivation to be active), indicating that outdoor loose parts play may be an effective strategy for promoting children's physical literacy. This is a novel and important finding, and one that requires further investigation.
